# Relationship between Plantar Pressure and Sensory Disturbance in Patients with Hansen’s Disease—Preliminary Research and Review of the Literature

**DOI:** 10.3390/s20236976

**Published:** 2020-12-06

**Authors:** Syoichi Tashiro, Naoki Gotou, Yuki Oku, Takahiro Sugano, Takuya Nakamura, Hiromi Suzuki, Nao Otomo, Shin Yamada, Tetsuya Tsuji, Yutaka Asato, Norihisa Ishii

**Affiliations:** 1 Department of Rehabilitation, National Sanatorium Tamazenshoen, Higashi-Murayama, Tokyo 189-0002, Japan; oku09078467915@gmail.com (Y.O.); atynakamura@gmail.com (T.N.); suzuki-hiromiaa@mhlw.go.jp (H.S.); 2 Department of Rehabilitation Medicine, Keio University School of Medicine, Shinjuku, Tokyo 160-8582, Japan; cxa01423@nifty.com; 3 Department of Rehabilitation Medicine, Kyorin University School of Medicine, Mitaka, Tokyo 181-8611, Japan; yamada-shin@ks.kyorin-u.ac.jp; 4 Department of Prosthesis and Orthosis, National Sanatorium Tamazenshoen, Higashi-Murayama, Tokyo 189-0002, Japan; 523goto@gmail.com (N.G.); sugataka1975.sawa@ymobile.ne.jp (T.S.); 5 Department of Rehabilitation, National Hospital Organization Tokyo Hospital, Kiyose, Tokyo 204-8585, Japan; 6 Department of Orthopaedic Surgery, National Sanatorium Tamazenshoen, Higashi-Murayama, Tokyo 189-0002, Japan; notomo@keio.jp; 7 Department of Orthopaedic Surgery, Keio University School of Medicine, Shinjuku, Tokyo 160-8582, Japan; 8 Department of Surgery, National Sanatorium Tamazenshoen, Higashi-Murayama, Tokyo 189-0002, Japan; asatoy-kkr@umin.ac.jp; 9 Department of Dermatology, National Sanatorium Tamazenshoen, Higashi-Murayama, Tokyo 189-0002, Japan; ishii-norihisa@mhlw.go.jp

**Keywords:** gait analysis, orthosis, insole, prosthesis, Hansen’s disease, leprosy, peripheral neuropathy, wound, precision rehabilitation

## Abstract

Orthoses and insoles are among the primary treatments and prevention methods of refractory plantar ulcers in patients with Hansen’s disease. While dynamic plantar pressure and tactile sensory disturbance are the critical pathological factors, few studies have investigated whether a relationship exists between these two factors. In this study, dynamic pressure measured using F-scan system and tactile sensory threshold evaluated with monofilament testing were determined for 12 areas of 20 feet in patients with chronic Hansen’s disease. The correlation between these two factors was calculated for each foot, for each clinical category of the foot (0–IV) and across all feet. A significant correlation was found between dynamic pressure and tactile sensation in Category II feet (*n* = 8, *p* = 0.016, r^2^ = 0.246, Spearman’s rank test). In contrast, no significant correlation was detected for the entire foot or within the subgroups for the remainder of the clinical categories. However, the clinical manifestation of lesion areas showed high variability: (1) pressure concentrated, sensation lost; (2) margin of pressure concentration, sensation lost; (3) pressure concentrated, sensation severely disturbed but not lost; and (4) tip of the toe. These results may indicate that, even though there was a weak relationship between dynamic pressure and tactile sensation, it is important to assess both, in addition to the basics of orthotic treatment in patients with Hansen’s disease presenting with refractory plantar ulceration.

## 1. Introduction

Hansen’s disease, also known as leprosy, is a chronic infectious disease caused by *Mycobacterium leprae*, which affects the skin, eyes and peripheral nerves [1]. Hansen’s disease initially causes peripheral nerve damage, including an acute immunological leprosy reaction, and then induces late-onset neuropathy in presumably immunological mechanisms. Thus, physical disability gradually progresses for decades even if the bacteria are eliminated from host [2,3]. Plantar ulceration is a clinically common and important complication among the chronic patients [4]. The pathophysiology of plantar ulcer is a multiplex of impairment, including tactile and proprioceptive sensory disturbance; paresis; balance impairments; autonomic nerve disorder; vision disorder; and joint, toe and limb deformity, e.g., Charcot joint, bone absorption and amputation [5,6,7]. Furthermore, the sequela include the wound infection, which can progress to phlegmon or myelitis and eventually lead to amputation or development of squamous cell carcinoma [6]. Thus, plantar ulcers are a serious health concern and a significant cause of disability in patients with Hansen’s disease [8].

The orthotic treatment is one of the core treatments for plantar ulceration. The pathogenesis of ulceration is characterized by: (1) disturbance in circulation due to pressure or sympathetic nerve disorder; (2) mechanical direct damage; (3) indirect mechanical damage via tissue inflammation; (4) infection; and (5) frostbite and burning [9]. Clinicians have focused on two major causes of mechanical damage accumulation: plantar pressure and sensory disturbance [5,6,10]. Since the mononeuropathy multiplex in Hansen’s disease form a patchy pattern and asymmetrical distribution of the impairments, which results in widely variable pathogenesis among individuals [6], it is challenging to conduct orthotic treatment [11]. The principle in designing orthoses is to depressurize areas of concentrated weight-bearing and protect vulnerable areas of the foot, together with a general method to check the deformity, callus or paresis of the foot closely [12,13]. However, there have been no qualified intervention studies from this standpoint. A recent Cochrane review [14] included only two studies on orthotic treatment in Hansen’s disease comparing campus shoes and polyvinyl chloride boots and molded double-rocker plaster shoe and below-knee plaster [15]. Neither study conveys the idea of detailed evaluation for plantar pressure or sensory assessment, while some researchers state the necessity of assessing sensory loss for shoes or orthosis design [11]. There is increasing evidence emphasizing the importance of dynamic pressure assessment [16,17,18] or a combination of both assessment types [19]. Thus, researchers are reaching a consensus on utilizing sensors in the precise treatment of plantar ulceration [6,20]. However, since only a few reports utilize multi-modalities including both assessment types, the relationship between plantar pressure and sensory disturbance is still not wholly delineated. Subsequently, it is difficult for clinicians to decide which parameter or both should be referred to in orthotic treatment of Hansen’s disease [19,21]. On these grounds, the purpose of the present study was to evaluate whether a relationship exists between dynamic pressure and sensory disturbance in Hansen’s disease upon the knowledge reported in previous literature.

## 2. Materials and Methods

### 2.1. Participants

The present study was performed as a retrospective observational study. We defined this study as a preliminary study since the number of subjects was relatively smaller than other similar studies. To determine this study’s sample size, we referred to two previous studies that assessed the relationship between sensory input and plantar pressure in healthy subjects with an artificial sensory disturbance model, which both assessed 20 feet in 10 participants [22,23]. In addition, the sample size was calculated using a Hansen’s disease research, which reported the standard deviation of plantar pressure about 200 kPa corresponding to 0.21 kg/cm^2^ in this study [6]. When setting σ as 2.1, δ as 1.0 and (1 − α) as 0.95, it was 19. Accordingly, the present study included 20 feet from 10 consecutive patients for whom both plantar pressure and tactile sensory assessments took place from April 2014 to March 2015 (Table 4). To avoid the selection bias, we started the inclusion from the first case who underwent both assessments. These patients were residents of the National Sanatorium Tamazenshoen (Higashi-Murayama, Japan) and provided written informed consent before this retrospective analysis. All participants received conventional dermatological care for treatment or prevention of plantar ulceration. Data of three out of 10 patients (Cases A–C) were partly published previously as a small case series in the Journal of Dermatology [19]. The protocol was in complete compliance with the ethical guidelines of the 1975 Declaration of Helsinki and was approved by the Ethics Committee in Tamazenshoen (No. 29-1).

### 2.2. Setting

The present study took place in a Hansen’s disease national sanatorium in Japan. While very few new cases were reported in Japan for decades, most of the ex-leprosy patients with sequelae live in one of thirteen national Hansen’s disease sanatoriums because of the social stigma, the presence of peer support and the easy access to specialists’ care. In total, there are 1090 residents with 86.3 average age (2020) in these sanatoriums.

### 2.3. Clinical Category

**Figure 1:**
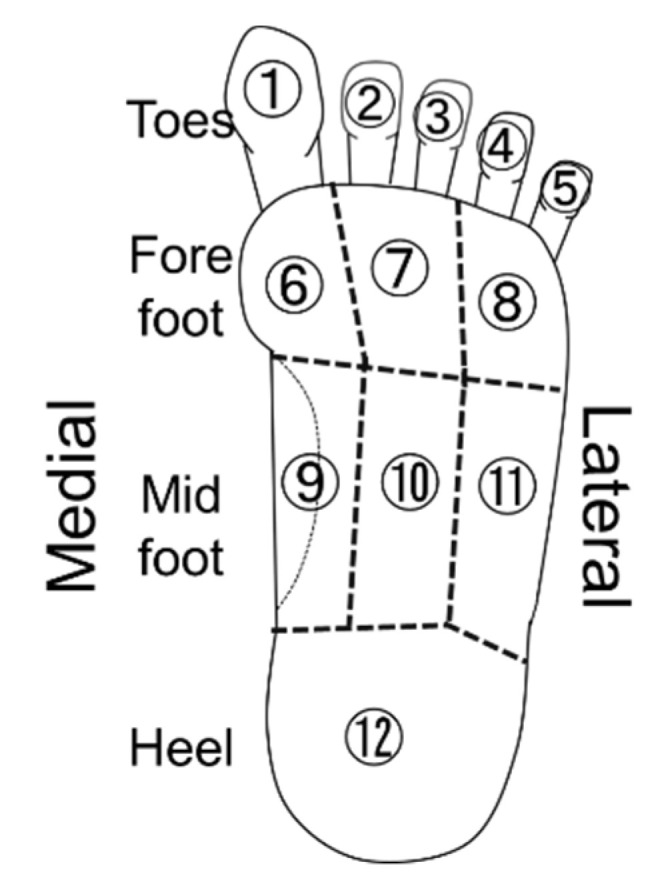
Plantar segments.

Dynamic plantar pressure and tactile sensation were assessed in 12 finely divided segments of the plantar surface to determine regions at a high risk of ulceration and provide a necessary yet sufficiently minimized insole, orthosis or prosthesis (Figure 1). This dividing method is developed from previous research with six divisions [5], which also investigated tactile sensation and plantar pressuring. The forefoot was divided corresponding to Toes 1, 2–3 and 4–5, and the midfoot was divided into arch, medial and lateral parts of weight-bearing area. Since the orthotic treatment naturally refers to the shape of the foot and motor function, we did not include these factors in the analysis.

Plantar areas were subsequently checked for present or past ulcerations by searching participants’ medical records within the past five years. Participants’ feet were then classified into one of the following five categories using a slightly modified version of a previous classification by Enna et al. [24] in 1976 under the principle of prevention of plantar ulcer. Although they focused on the ulcer itself, we included status which may lead to ulceration according to previous reports [6,25]. We modified two points: replaced the use of “sensory loss” with “sensory disturbance” and focused not only on scarring but also on recurrent callus formation, which easily leads to callous ulcers typically forming underneath [3,26]. Accordingly, we applied the following classification: Category 0, normal foot without remarkable sensory disturbance; Category I, grossly normal foot without scarring or status leading to scarring, persistent or recurrent injury, crack and callus/corn formation, but with a decline of sensation; Category II, grossly normal foot with scarring or status leading to scarring, persistent or recurrent injury, crack and callus/corn formation; Category III, foot with at least one deformity that does not affect either the length or the width of the foot; and Category IV, pathologic short or narrowed foot due to bone absorption or amputation. Modifications in classification are summarized in Table 1. We considered these modifications necessary to adjust to the current clinical practice, reflecting treatment development, the paradigm shift in clinics from cure to ulcer prevention and the transition in patients’ profiles that proportion of severe cases is markedly decreasing [1,3] (Table 1).

Twelve segments of the plantar surface of the foot are schematically shown. Both dynamic pressure and tactile sensory perception were analyzed and assessed for each segment: the toes; medial, middle and lateral regions of fore- and mid-foot; and the heel.

### 2.4. Sensory Assessment

The finer limit of the tactile sensory threshold was measured using a Semmes–Weinstein monofilament test (Sakai Medical. Co. Ltd., Tokyo, Japan) on 12 areas on the foot plantar surface. Briefly, the monofilaments were applied perpendicularly to the center of the corresponding region three times by the same examiner. Sufficient force was applied until the filament bent or twisted. One or more false examination(s), in which the filament was not actually touched on the skin, were included in addition to three measurements. If the participant gave incorrect answers more than once in the three measurements, we judged the filament strength below the participant’s sensory threshold. Average values were recorded with the participant in the supine position with their eyes closed [27]. For correlation analyses performed using participants’ actual values, sensory thresholds were classified into five levels: (1) preserved protective sensation, perceptible of <2 g filament; (2) diminished protective sensation, perceptible of 2 g filament; (3) loss of protective sensation, perceptible of 10 g filament; (4) residual deep pressure, perceptible of 300 g filament; and (5) sensory loss, no reaction with any filament [28,29]. In the context to promote a practical method to combine with plantar pressure assessment, we only applied tactile sensation as in a previous study [5].

### 2.5. Measurement of Dynamic Plantar Pressure with the F-Scan System

Dynamic pressure was measured using an F-scan II system (Tekscan, Boston, MA, USA), which comprises a foot-shaped thin in-shoe pressure sensor sheet that the examiner can optimize (i.e., cut to size). The apparatus determines the distribution of plantar pressure by monitoring the liquid’s status inside the sensor sheet. Briefly, this system consists of two ultra-thin polyester film layers on which electrically conductive ink is printed in a rows-columns manner. It is coated with a pressure-sensitive resistive ink that allows electrical signal output according to the pressure loading, and then it enables pressure quantification. The sensor sheet cables emerge from the medial side of the feet and return to the PC via the participant’s lumbar belt to avoid disturbing their motion [30]. The in-shoe-mat design enables entire plantar pressure measurement inside shoes and orthoses. While pressure–time integral [16,18] or pressure contact ratio [20] were also sometimes used, peak plantar pressure was solely assessed in this study as in a previous report [6]. It is because an increasing number of reports suggest the causal relationship between abnormal peak pressure and ulcer development in diabetes [31,32]. Patients were asked to make a straight gait of more than 10 m at the participants’ self-selected speed without any ortho-prosthetic devices [33]. The peak plantar pressure was determined along with the principle of midgait protocol [34]. Briefly, plantar pressure during a 10-m gait was recorded twice. After removing the first and last ambulatory cycle, the peaks were calculated from each trial. The averaged values were recorded. In addition, participants wearing orthosis were further analyzed with a total-foot ankle-foot orthosis (e.g., the right foot of Case B). The highest values were plotted for each cell and reconstructed into the figures of pressure distribution, i.e., the figure does not represent a certain time-point. While pressure–time integral is another fundamental value in plantar pressure analysis, it shows a high concordance with peak pressure [35]. Therefore, dynamic pressure was only applied in this study to generalize this assessment among clinicians in this field.

### 2.6. Analysis of the Relationship between Dynamic Pressure and Tactile Sensory Disturbance in Hansen’s Disease

The correlation between peak plantar pressure (kg/cm^2^) and tactile sensory ability, as shown in 1/threshold (1/g) at each plantar region, was assessed using Spearman’s rank test. We tested whether patients demonstrated a positive correlation, whereby the dynamic pressure is higher in areas with finer tactile sensory function. We assessed the correlation using three different methods: individual analysis for 20 feet with 12 different areas; five subgroup analyses classified according to the clinical severity category [24]; and analysis with the clinical data across the entire foot.

### 2.7. Literature Review

The literature on the pathogenesis of plantar ulceration utilizing plantar pressure measurement and that on the orthotic treatment for plantar ulceration were searched on PubMed, using the following tags: Hansen’s disease, leprosy, plantar, pressure, ulcer. Articles were searched since 1980, it was determined by abstract whether to include them.

## 3. Results

### 3.1. Review of Literatures

Biomechanical sensor plays an important role in the precise assessment of the pathology of plantar ulceration. However, Hansen’s disease, the number of researches on plantar pressure using sensors is limited, and most of them are observational studies. (Table 2 and Table 3).

### 3.2. Clinical Features

#### 3.2.1. Clinical Features of Cases

- Significant correlation between tactile sensory threshold and dynamic pressure were observed in three feet with Category II (C-left, *p* = 0.038; r^2^ = 0.626: D-right, *p* = 0.038; r^2^ = 0.626: and E-right, *p* = 0.017; r^2^ = 0.717).
- Refractory ulcers were not always observed in areas with overlapping sensory deficits and high weight-bearing.
- Cases with higher category tend to need orthoses, while most cases with Category II feet needed insole. There was no need for orthotic treatment for feet with categories less than Category II.

**Figure 2:**
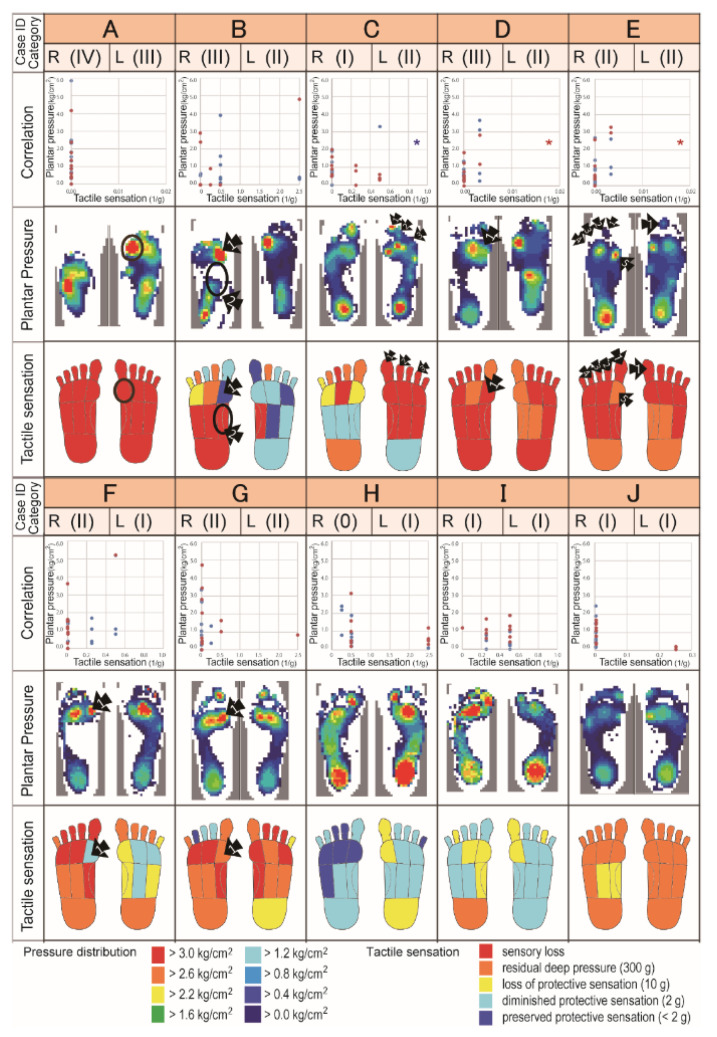
Clinical feature of each foot. The present or past ulceration, tactile sensory threshold, dynamic pressure, and correlation between sensation and dynamic pressure are summarized.

The clinical features, present or past ulceration, tactile sensory threshold and dynamic pressure are summarized in Figure 2. (see also Table 4).Together, the actual dynamic pressure values in each plantar area are presented in Table S1. Interestingly, refractory ulcers were not always observed in areas with overlapping sensory deficits and high weight-bearing. The profiles of areas where plantar ulcers develop were characterized by: (1) pressure concentrated sensation loss; (2) margin of pressure concentration, sensation lost; (3) pressure concentrated, sensation severely disturbed but not lost; and (4) tip of the toe, but these conditions were not sufficient or necessary in forming ulcers. The relationship between dynamic pressure and tactile sensation in each of the 20 feet was independently calculated (Figure 2, second row of cases), and a significant correlation was found in three feet (C-left, *p* = 0.038; r^2^ = 0.626: D-right, *p* = 0.038; r^2^ = 0.626: and E-right, *p* = 0.017; r^2^ = 0.717).

#### 3.2.2. Treatment Details for Each Subject

**Figure 3:**
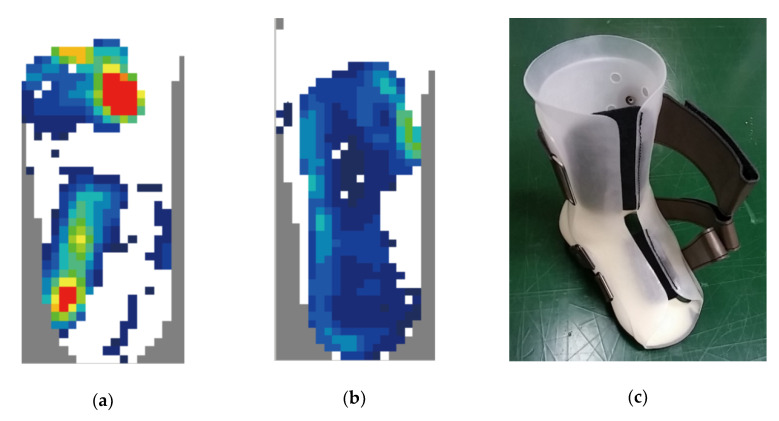
Depressurization with precision orthotic treatment in Case B. Dynamic plantar pressure without (**a**) and with (**b**) orthosis is shown. The pressure distribution became equalized over the entire plantar surface. (**c**) A picture of orthosis.

Cases A and B were treated with rigid type depressurization orthoses. A partial forefoot orthosis was prescribed for Case A due to severe upper extremity dysfunction. A plastic ankle-foot orthosis (pAFO) with a double-layer structure was provided for Case B, made with a soft inner layer and a hard outer layer, enabling a firm fixation without excessive mechanical stress onto a specific area. Cases C–F were treated with an insole or insole and partial protective orthosis, whereas Case G refused any orthotic treatment. No orthotic intervention was done in Cases G and H because neither wounds nor sensory deficits were observed. All ulcers could be controlled, except for Case G, who refused treatment. Although it is only an observatory result, our precision orthotic approach successfully treated or prevented plantar ulcerations. The treatment courses of Cases A–C were previously described as a case series in detail [19]. We further assessed the dynamic pressure of this foot while wearing the orthosis with the F-scan system. The analysis demonstrated good depressurization on the plantar surface of the foot with the orthosis (Figure 3).

Clinical features of sensory function and dynamic pressure are shown. In the first row, case ID (Cases A–J), side of the foot and impairment Categories 0–IV are shown. In the second row, the correlation between plantar pressure and sensory threshold for each foot is shown. Blue dots and lines represent values of the left feet, while red dots represent values of the right feet. A significant correlation was observed in the right foot of Case C, and the left foot in Cases D and E (*p* < 0.05). In the third row, the results of the plantar pressure analysis are shown. For pressure measurements, the stronger is the color, the higher is the pressure loading, that is red-orange-yellow-green-blue-white in order from strongest to weakest. In the fourth row, the tactile sensation schemes are shown. The sensory threshold was classified into five levels: (1) preserved protective sensation, perceptible of <2 g filament; (2) diminished protective sensation, perceptible of 2 g filament; (3) loss of protective sensation, perceptible of 10 g filament; (4) residual deep pressure, perceptible of 300 g filament; and (5) sensory loss, no reaction with any filament. The stronger is the color, the more severe is the disturbance, that is red-orange-yellow-light blue-blue in order from strongest to weakest. Black circles and arrowheads indicate areas or points where a wound was or had been observed. Similar distribution maps of plantar pressure and sensory function of Cases A–C were published in a previous case report [19].

### 3.3. The Relationship between Plantar Pressure and Tactile Sensory Function

- A significant correlation between dynamic plantar pressure and tactile sensory threshold was observed in Category II feet (*n* = 8, *p* = 0.016, r^2^ = 0.246, Spearman’s rank test), indicating patients in this category tend to bear their body weight at an area where the sensory function is preserved.
- No correlation was detected between tactile sensory threshold and dynamic pressure in both severer and milder impairment other than clinical Category II.

**Figure 4:**
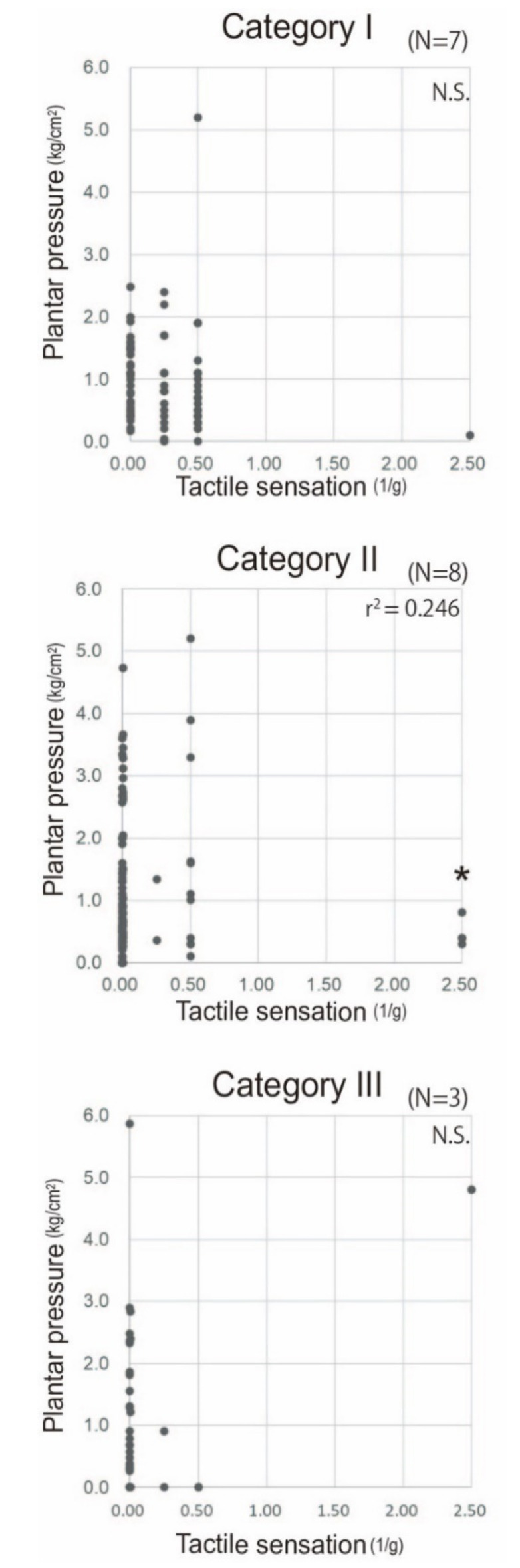
Relationship between dynamic pressuring and sensory function according to the severity of sensory disturbance; The correlation between dynamic plantar pressure and tactile sensory threshold is presented for each clinical category of Hansen’s disease in which more than one foot was classified (Category I, *n* = 7, grossly normal foot without scarring, but with decline of sensation; Category II, *n* = 8, grossly normal foot with scarring or callus commonly at the ball of the foot; and Category III, *n* = 3, foot with deformity that does not affect either the length or the width) [24]. A significant correlation was observed in Category II (*p* = 0.016, r^2^ = 0.246), while no significant correlation was observed in the other categories (*p* > 0.05).

Next, we reassessed whether patients bear weight at the area where sensory function was preserved across all cases and within the five subgroups classified according to the modified version of the clinical neuropathic foot categories [24]. No correlation was detected between dynamic plantar pressure and tactile sensory threshold across all cases (*p* > 0.05, r^2^ = 0.024, data not shown, Spearman’s rank test). In the subgroup analyses, while two factors did not show significant correlations in Categories I and III (Category I [n = 7]: *n*.s.; Category III [*n* = 3]: n.s.; Spearman’s rank test), a weak but significant correlation was observed in Category II feet (*n* = 8, *p* = 0.016, r^2^ = 0.246, Spearman’s rank test). One foot each that did not show a correlation was classified into Categories 0 and IV (Figure 4).

## 4. Discussion

### 4.1. A Concise Review of the Literature

The plantar pressure measurement can play a vital role in treating Hansen’s disease. While a method utilizing a few transducers was first introduced by Bauman et al. in 1963 [39,40], Sabato et al. utilized a platform-type sensor to investigate the mechanism of plantar ulceration in the early 1980s [5]. While such a stationary type device is still used even in the assessment of dynamic pressuring [21], researchers should pay attention to that evaluation of two steps at least is needed for the qualitative assessment [34]. Current in-shoe sensor systems enable assessing both static and dynamic plantar pressure [37,41,42] and even daily cumulative stress [37]. Dynamic pressure analysis is an assessment that better evaluates the primary function of the foot [43,44] but can assess the specific features of walking, including sliding, friction, shear forces and thrust [39]. Notably, there has been almost no study investigating the differences among clinical subgroups. To our knowledge, only Condeiro et al. compared multibacillary (MB) and paucibacillary (PB) leprosy: while the loss of protective sensibility was predictive in MB patients, the plantar pressure peaks seemed more critical in PB patients [34]. Besides, there seems no study reporting the difference corresponding to the severity of foot impairment.

The clinical application of plantar pressure assessment is still limited in the clinics of Hansen’s disease [16,19,24,45]. Instead of assessing dynamic pressure, clinicians typically assess sensory function in addition to the ordinal checkpoints, such as deformity, bone process, paresis, balance and gait pattern [11,12,13]. While just a case series and a preliminary report have suggested a clinical strategy utilizing both plantar pressure and sensory assessments [16,19], to date, there has been a shortage of scientific evidence demonstrating the importance of this strategy. Another minor issue is the way to divide the plantar foot. Although it is not totally confirmed, 10 divisions seems most widely applied especially in plantar pressure measurement [20,46]. Besides, 6 [5,21,34], 4 [6] and 12 divisions [19], which were developed based on the six-division method, were also applied. Finer separation might be better to determine regions at a high risk of ulceration and to provide a necessary yet sufficiently minimized insole, orthosis or prosthesis.

On these grounds, it would be necessary to determine the relationship between plantar pressure and sensory function with finely divided plantar assessment in patients with Hansen’s disease to better perform the orthotic treatment.

### 4.2. Discussion on the Current Study

The present study firstly investigated the relationship between dynamic plantar pressure and tactile sensory function according to the clinical category of Hansen’s disease and found a correlation in patients with clinical Category II disorder, who are at risk of developing plantar ulceration. In other words, those patients demonstrated a significant trend to bear body weight in plantar areas where the sensory function is relatively preserved. This result is consistent with a previous study on healthy participants with an ice exposure-induced sensory disturbance model, which evaluated a similar sensory decline pattern [22]. This phenomenon may prevent plantar ulceration at sensory deficit sites, where the defensive reaction is absent. Simultaneously, it would partially explain the pathogenesis by which ulcerations developed in areas without sensory loss. We consider the present result will help design the orthosis or insoles without apparatus to assess plantar pressure. However, it is noteworthy that the areas with plantar ulcers showed remarkable variability, and they do not always develop at sites with peak pressure loading, as indicated in previous studies [5,19]. Therefore, we would like to recommend applying these two specific assessments to the necessary observation even in this clinical grade. On the other hand, no relationship was found in the other clinical grades, namely Categories 0, I, III and IV. Therefore, whereas many progressive studies in this field only performed one out of these two evaluations [11,16,17,18,20,36], it would be better to apply both assessments. In patients with severer impairments, it might be because the patients can apply a limited area of the plantar foot to manage their functional ambulation with maintaining balance and avoiding excessive pressure due to severe functional deficit and deformity. Besides, there would be no need to do so for the feet with milder impairment.

It is essential to provide personalized and appropriate orthoses for patients with Hansen’s disease [13,19]. Simultaneously, clinicians should be aware of the medical expertise required and patients’ physical difficulty in correctly equipping the orthosis, particularly in patients suffering from severe visual and hand dysfunction [3]. The dependence on properly wearing their orthosis restricts patients’ daily activity. Therefore, the greater does *not* serve as the lesser concerning the orthotic treatment in patients with Hansen’s disease, but it often goes the easier, the better [19,47]. These principles emphasize the importance of detailed assessment utilizing the biomechanical sensors to figure out the area at risk of ulceration and provide a best-suited orthosis. Thus, sensors are indispensable to proceed precision orthotic treatment that will help patients perform activities of daily living and improve their quality of life.

Foot care, including treatment of refractory plantar ulceration in patients with Hansen’s disease, requires not only highly specialized wound care but also highly specialized rehabilitative treatments. It is sometimes difficult for elderly patients to get accustomed to wear the orthosis and to move or live with it. Therefore, a rehabilitative intervention is required to prevent disuse deterioration of bodily functions and promote to learn good gait and basic activities, particularly with an orthosis [47]. Thus, a multidisciplinary, personalized approach involving dermatologists, prosthetist–orthotists and rehabilitation specialists is important in the treatment of patients with Hansen’s disease [1,48].

### 4.3. Perspectives for Precision Orthotic Treatment Utilizing Sensors

Dues to the shortage of studies on plantar ulceration in Hansen’s disease, the orthotic treatment is highly dependent on the experience of prosthetist–orthotist. One reason is that the number of patients is rapidly decreasing in non-epidemic countries, which are often developed countries where clinical research is well taken. On the other hand, increasing research is promoting understanding and countermeasures for plantar ulceration in the field of diabetes. We consider it crucially important to put following new methods into perspective for clinics and researchers of Hansen’s disease to improve the treatment.

Many patients with diabetes mellitus experience plantar ulceration because it causes mixed fiber neuropathy including motor, small and large sensory fibers for touch, vibration and proprioception and autonomic nerves as in Hansen’s disease [3,49]. It is known the plantar ulcerations derived from these two different diseases exert resembling features in terms of healing rate and time required [50] and physical and psychosocial aspects [51]. Nonetheless, there seem some differences between these patients. Firstly, diabetic patients generally suffer from more co-morbidities [6]. Secondly, leprosy patients face severe stigma and disease ignorance, in both epidemic and non-epidemic countries. These backgrounds may lead to weak social support and limited treatment environments in the majority of countries [1,52,53]. Lastly, the disease pathophysiology is different in some aspects. While diabetic neuropathy is characterized by distal symmetric polyneuropathy [54], Hansen’s disease induces asymmetric multiple-mononeuropathies [3]. In addition, the presence of peripheral artery disease and tissue glycosylation in diabetes will influence the damage and response of the foot against mechanical stress [6,55]. Even though the phenotype and treatment course are relatively similar, such fundamental pathological differences may affect the formation process and countermeasure of plantar ulceration. On these grounds, we consider the research on the two diseases should be done independently.

Systems for plantar pressure measurement can be classified into two major types, platform systems and in-shoe systems [41,42], and many new devices are being introduced, particularly from diabetes research [56]. The F-scan [17,19] or Pedar in-shoe system (Novel GmbH, Munchen, Germany) [37] and the EMED^®^ pressure distribution platform (Novel GmbH) [6,23] are the most used devices in the clinics for patients with Hansen’s disease. Since the F-scan system enables measuring pressure distribution in the shoe, insole, orthosis and prosthesis [42], it was best suited for the current study. While the current study only assessed peak pressure, increasing evidence establishes the state-of-the-art to refer to other variables that can be recorded by these devices as well. Pressure–time integral represents the cumulative effect of mechanical stress over time [16]. Although a research group suggested this parameter has a concordance with peak plantar pressure [35], it is reported showing a better significance in the area with ulceration [57]. The plantar contact area while wearing orthosis was the other parameters applied in a previous study together with pressure–time integral [18]. This parameter is especially important in designing an orthosis to avoid pressure on the vulnerable area. However, we did not check it as a routine assessment because it was manually evaluated in manufacturing orthosis. In contrast, we specifically checked this value in Case B, for whom a hard type ankle-foot orthosis was provided. Peak vertical ground reaction force is another relatively new parameter that reflects the subjects’ muscle power and shape of foot and claw [58,59]. While there have been no studies for Hansen’s disease on this parameter, a further investigation would be needed to reveal its relationship with ulceration, especially on the toes.

Applying the newly developed wireless foot plantar pressure system will be useful for more accurate, reliable and safe measurement and monitoring of plantar pressure in daily life [42,60,61]. As discussed in previous reports, monitoring plantar pressure across an entire day enables clinicians to capture critical gait and standing habits that may lead to scar generation. It will also be useful in the prevention of falling [62,63]. The importance of daily monitoring is particularly true in some Asian countries, where people tend to spend most of their lives on their feet. Currently, a technology to measure plantar pressure in socks is being introduced [64]. This system may have advantages over the in-shoe system in terms of preventing in-shoe sensor slip [42] and measuring pressure on the side of the foot. Due to severe deformity, some patients cannot help bearing weight on the lateral surface of the foot or thigh, particularly in cases with amputation, causing refractory plantar ulceration. A combination with other assessment modalities will be informative in these cases [65,66,67]. In particular, researchers have established an accurate method utilizing musculoskeletal foot modeling and finite element 3D subject-specific modeling to predict ulcer risk in the field of diabetes. The application of this newly developed method will open further prospects for the treatment of Hansen’s disease [68,69].

### 4.4. Limitations

The present study has some limitations. First, this study only applied a few factors. In sensory assessments, we only focused on the tactile sensation because it has been shown to induce an escape reaction [22,23] and has the most satisfactory ability to distinguish the area receiving pressure [6,11,19] for the prevention of plantar ulceration. However, this would require further investigations because other sensory modalities such as proprioception or vision influence plantar pressuring and gait. Regarding the plantar pressure, we only evaluated the peak pressure while pressure–time integral [16,18] or pressure contact ratio [20] of dynamic pressure and static pressure [37] have also been applied in Hansen’s disease studies. In addition, there may be other important factors bridging plantar pressure and sensory disturbance, such as gait kinematics, joint deformity and paresis [70], and the third factor might more affect the feature of ulceration. Therefore, researchers still need to keep a broad perspective on ulcer mechanisms, and studies of multi-factor investigation are needed. Second, most of the feet in this study were classified as denervated in Category I or II. Therefore, these results may not represent the entire population of patients with Hansen’s disease. In addition, the sample size of feet was small for subgroup analysis. In addition, while this study had the same number of samples as the previous studies, 20 feet among ten subjects is not equivalent to 20 feet in 20 subjects, which might be a statistical problem of this preliminary study. A more extensive study, particularly one focusing more on severe cases, is needed. Third, we applied a disease classification that is a modified version of a previous classification method [24]. Validation of this new classification is required. Besides, the method to divide the plantar surface applied in this study is non-validated. Fourth, while we only evaluated plantar pressure in Case B just after donning the orthosis, the time transition assessment would give us further insight, as was investigated in a previous report [71]. Fifth, it is a non-controlled intervention as the orthotic treatment. A further intervention study to compare orthotic treatment with and without detailed assessment with plantar pressure and sensation will be necessary. Lastly, while the “microclimate” of the plantar surface of the foot is focused on in the field of diabetes foot research, many of its elements (i.e., shear stress, temperature and humidity) were not considered [72,73]. Since there have been no reports, to the best of our knowledge, applying this principle in the field of Hansen’s disease, further investigations will be fruitful. For instance, it is relatively easy to measure shear stress using a similar apparatus [74].

## 5. Conclusions

While the present study revealed a significant trend that patients with Category II neuropathic foot avoid weight bearing in the area of the foot with sensory loss, the relationship was weak and no correlation was found in the feet in the other clinical categories. Moreover, the profiles of dynamic pressure and tactile sensory disturbance at lesion areas were highly diversified. Therefore, we conclude that it will be indispensable to assess both dynamic pressure and tactile sensation, together with the established basics of orthotic treatment, to provide appropriate orthoses for refractory plantar ulceration in patients with Hansen’s disease. Together with the comparison of various parameters of plantar pressure, and the application of newly developed methods particularly from diabetes research, future investigations are required to delineate the detailed features of plantar ulceration and establish a systematic methodology for designing precision orthotic treatment in patients with Hansen’s disease.

## Figures and Tables

**Table 1:** Clinical categories of neuropathic foot in Hansen’s disease.

Category	Tashiro et al. (2020)	Enna et al. (1976)
0	Normal foot without remarkable sensory disturbance	Normal foot without remarkable sensory loss
I	Grossly normal foot without scarring or status leading to scarring, persistent or recurrent injury, crack and callus/corn formation, but with decline of sensation	Grossly normal foot without scarring, but with loss of sensation
II	Grossly normal foot with scarring or status leading to scarring, persistent or recurrent injury, crack and callus/corn formation	Grossly normal foot with scarring commonly at the ball of the foot
III	Foot with deformity that does not affect either its length or width	Foot with deformity that does not affect either its length or its width
IV	Pathologic short and/or narrowed foot due to bone absorption or amputation	Pathologic short and/or narrowed foot due to bone absorption or amputation

**Table 2:** Research on the pathogenesis of plantar ulceration utilizing plantar pressure measurement in Hansen’s disease.

Author Year Country Study Type	Subject Number Number of Areas Purpose of Study	Items Assessed	Findings
Sabato [5] 1982 Israel Observation	30 patients 6 areas To determine factors related with ulcer	Sensation (pin-prick), Dynamic pressure (ground pressure pattern), Active range of motion of ankle	Sensation and ground pressure are associated with ulcer, while sensation and pressure showed relationship No relationship with ankle function
Greve [36] 1994 Brazil Observation	13 patients, 17 control 2 areas To determine factors related with ulcer	Static pressure	Asymmetry and increased pressure were associated with plantar ulcer
Bhatia [20] 1999 India Observation	108 patients, 52 control 10 areas To determine factors related with ulcer	Dynamic peak pressure (normalized), Pressure contact ratio, comorbidity (claw-toe, bone change, foot drop)	Dynamic foot pressure was higher in patients and associated with high incidence of ulcers.
Slim [6] 2012 Netherlands Observation	39 patients 4 areas To determine factors related with pressure and walking capacity	Dynamic pressure, age, weight, Sensation (pressure, vibration), toe-foot deformity, joint mobility, ankle muscle strength and callus.	Highest pressure is associated with sensation, toe amputation/absorption and hallux valgus are useful to find the risk of excessive pressure. Foot impairments independently affect reduced walking capacity.
van Schie [37] 2013 Netherlands Observation	39 patients (9 with current, 15 with previous, 15 without ulceration No segmentation To determine factors related with ulcer	Barefoot peak pressure, in-shoe peak pressure and daily cumulative stress with a specific device	Current and previous ulceration do not differ on barefoot pressure. In-shoe peak pressure increase in persons with current ulceration, who were less active, resulting in no difference in daily cumulative stress.
Condeiro [21] 2014 Brazil Observation	51 patients (MB type 31; PB type 20), 20 control 6 areas To investigate the influence of leprosy type on ulceration	Sensation (tactile sensation with mono-filament test), Baropodometer, Dynamic pressure	Loss of protective sensibility in MB patients is predictive of plantar ulcers Plantar pressure peaks seem to be of greater importance in PB patients in ulcer prediction

**Table 3:** Research on the orthotic treatment for plantar ulceration in Hansen’s disease.

Author, Year Country, Study Type	Subject Number Number of Areas Purpose of Study	Items Assessed	Findings
Birke [17], 1994 UK, Observation	10 patients (6 for orthotic treatment) To compare shoes-sandal vs. extradepth shoe vs. Barefoot	dynamic pressure	Peak pressure was lower with Bombay sandals, the Chinese tennis shoe, the extradepth shoe with an insole and the patients′ prescribed shoe
Cross [11], 1995 India, RCT	71 patients To evaluate effect of custom-made orthosis	Sensation (pinprick and vibration), Deformity	Wound healing was facilitated by orthotic treatment
Linge [16], 1996 Belgium, Preliminary Case	To find the way to reduce plantar pressure with insole	in-shoe dynamic pressure measurements for peak pressure and pressure–time integral	A shank to control insole rigidity reduced the overall peak pressures A deep canvas shoe with double-thickness insole is advantageous
Tang [18], 2015 Taiwan, Observation	8 patients To evaluate the effect of custom-made shoes and insole	dynamic pressure area, peak plantar pressures, contact time, pressure time integral with and without custom-made shoes	custom made shoes and total contact insoles were effective in increasing contact area and decreasing peak pressure in plantar surfaces
Rai [38], 2016 India, Intervention	17 patientsTo investigate effect of total contact cast	Sensation testing with 10-G monofilament	80% of the cases healed within 8 weeks
Tashiro and Oku [19], 2019 Japan, Case Series	3 patients 12 areas of foot To manufacture custom made orthosis	Sensation (tactile sensation with mono-filament test), Dynamic pressure	Coincidence of sensory loss and dynamic pressure is sufficient but not necessary condition in developing plantar ulceration.

**Table 4:** Patient characteristics and result of correlation analysis.

ID	Age (years)	Gender	Side	Steps Analyzed	*p*-Value	r^2^
A	80	F	Right	6	0.281	0.325
			Left	6	Non-calculated	
B	71	F	Right	8	Non-calculated	
			Left	8	Non-calculated	
C	79	M	Right	6	Non-calculated	
			Left	6	0.038	0.626
D	82	F	Right	6	0.038	0.626
			Left	6	0.271	0.332
E	87	M	Right	6	0.017	0.717
			Left	6	0.104	0.489
F	71	M	Right	6	0.602	0.157
			Left	6	0.618	0.150
G	68	F	Right	8	0.472	0.217
			Left	8	0.719	0.108
H	79	F	Right	10	0.876	0.047
			Left	10	Non-calculated	
I	87	F	Right	6	0.940	0.023
			Left	6	0.520	0.194
J	88	F	Right	6	Non-calculated	
			Left	6	Non-calculated	

Note: Patient characteristics and details of single-feet correlation analysis between dynamic pressure and tactile sensory function are shown.

## Supplementary Materials

The following are available online at https://www.mdpi.com/1424-8220/20/23/6976/s1, Table S1: Maximum planar pressure measured in each area.
