# Supplementary material for: Relationship between Plantar Pressure and Sensory Disturbance in Patients with Hansen’s Disease—Preliminary Research and Review of the Literature
Source: Sensors (Basel). 2020 Dec 6;20(23):6976. doi: 10.3390/s20236976 (PMC7730212; doi:10.3390/s20236976)
Supplement: Supplementary file 1 [file sensors-20-06976-s001.pdf]

sensors

MDPI

Supplementary Materials

Table S1. Maximum planar pressure measured in each area.

		Plantar Pressure (kgf/cm2)											
		①	②	③	④	⑤	⑥	⑦	⑧	⑨	⑩	⑪	⑫
A	Lt	0.3	0.6	0.4	0.3	0.1	3.9	1.1	0.4	0.5	0.4	0.3	1.6
	Rt	0.0	0.0	0.0	0.0	0.0	4.8	2.4	0.9	0.0	0.0	0.0	2.9
B	Lt	2.5	0.9	0.7	0.7	0.0	5.9	2.4	1.3	1.8	0.3	2.3	1.6
	Rt	0.4	1.1	0.4	0.6	0.0	2.4	2.4	1.8	0.3	1.4	4.2	0.9
C	Lt	1.9	0.7	0.0	0.0	0.0	2.0	2.0	1.3	0.5	0.8	0.5	3.3
	Rt	1.6	0.6	0.5	0.9	0.0	1.1	1.1	0.8	0.3	0.6	0.4	2.0
D	Lt	0.3	1.3	0.9	0.6	0.5	3.7	0.6	0.3	0.7	3.1	0.7	1.4
	Rt	1.2	0.8	0.3	0.5	0.6	1.3	2.8	0.3	0.0	0.4	0.4	1.9
E	Lt	1.0	0.1	0.2	0.2	0.1	2.7	1.4	0.5	1.0	0.6	0.9	2.6
	Rt	0.4	0.0	0.0	0.0	0.4	3.0	2.6	0.6	0.4	0.6	1.1	3.3
F	Lt	0.4	0.4	1.4	1.6	0.4	1.7	5.2	1.1	0.3	0.8	1.1	1.5
	Rt	1.2	1.5	0.3	1.6	0.8	5.2	3.6	1.1	0.0	0.0	0.9	1.5
G	Lt	2.7	1.4	1.0	0.3	0.4	3.3	2.7	0.8	0.0	0.4	0.4	1.3
	Rt	3.5	1.0	1.6	0.8	0.5	4.7	2.8	0.7	0.0	0.5	0.3	2.0
H	Lt	2.2	0.5	0.5	0.2	0.1	0.8	1.9	0.5	0.4	0.9	0.7	2.4
	Rt	3.1	0.3	0.5	0.2	0.4	0.5	1.2	0.6	0.5	1.0	0.6	1.6
I	Lt	0.8	0.3	0.2	0.0	0.0	0.5	1.0	0.4	0.3	0.3	0.3	1.9
	Rt	1.9	1.1	1.1	1.1	0.4	0.9	1.7	1.3	0.6	0.7	0.7	1.2
J	Lt	1.5	0.6	1.0	0.6	0.5	2.5	1.5	1.1	0.2	0.2	0.4	1.9
	Rt	1.7	0.8	0.8	1.1	0.4	1.5	1.1	1.2	0.0	0.2	0.3	1.5
